# ALKBH5 modulates macrophages polarization in tumor microenvironment of ovarian cancer

**DOI:** 10.1186/s13048-024-01394-4

**Published:** 2024-04-18

**Authors:** Yuanyuan An, Hua Duan

**Affiliations:** Gynecological Mini-Invasive Center, Beijing Obstetrics and Gynecology Hospital, Capital Medical University, Beijing Maternal and Child Health Care Hospital, 17 Qihelou Street, Beijing, 100006 China

**Keywords:** Ovarian cancer, ALKBH5; IGF2BP2, Macrophage polarization, Immune microenvironment, Single-cell

## Abstract

**Background:**

Macrophages play an essential role in regulating ovarian cancer immune microenvironment. Studies have shown that m6A methylation could influence immune microenvironment in cancer. In this study, we investigated the *roles* of m6A demethylase ALKBH5 and m6A recognition protein IGF2BP2 played in regulating macrophages polarization in ovarian cancer.

**Methods:**

In this study, we first explored the differentially expressed m6A methylation enzymes in M0 and M2 macrophages according to two independent GEO datasets. TIMER2.0 and GSCA database were used to explore the immune analysis of ALKBH5 and IGF2BP2 in ovarian cancer. K-M plotter and TIMER2.0 databases were used to evaluate the prognostic role of ALKBH5 and IGF2BP2 in ovarian cancer. For CNV mutation analysis of ALKBH5 and IGF2BP2, cBioPortal and GSCA databases were used. For single-cell analysis, sc-TIME and HPA softwares were used to analyze the roles of ALKBH5 and IGF2BP2 played in immune cells in ovarian cancer. To identify the role of ALKBH5 played in macrophage polarization, RT-PCR was used to verify the macrophage polarization related markers in vitro study. The function of ALKBH5 played in ovarian cancer was further analyzed through GO and KEGG analysis.

**Findings:**

In this study, we found that ALKBH5 and IGF2BP2 were up-regulated in M2 macrophages, which showed closely correlation with immune cells expressions in ovarian cancer, especially with macrophages. Ovarian cancer patients with higher expression of ALKBH5 and IGF2BP2 showed worse prognosis, possibly because of their close correlation with immune response. ALKBH5 also correlated with macrophage phenotypes in single-cell levels analysis. However, the expression level of IGF2BP2 in ovarian cancer immune microenvironment was very low. The results of RT-PCR indicated the potential role of ALKBH5 in M2 polarization of macrophages.

**Interpretation:**

ALKBH5 participated in regulating macrophage M2 polarization in ovarian cancer immune microenvironment.

**Supplementary Information:**

The online version contains supplementary material available at 10.1186/s13048-024-01394-4.

## Introduction

Ovarian cancer is one of the most common gynecologic malignant tumors, with a relatively high mortality rate. The statistics showed that 5-year survival rate of ovarian cancer patients was less than 44% [1]. The first-line treatment for ovarian cancer remains tumor cell reduction surgery combined with post-operative chemotherapy. However, the recurrence rate of ovarian cancer is as high as 70%. Studies have shown that tumor microenvironments (TME) participated in regulating the immune response and inflammatory response of cancer through various mechanisms [2–4]. Therefore, immunotherapy targeting TME has attracted extensive attentions.

TME refers to the tumor microenvironment which surrounds and nourishes the tumor cells, including blood vessels, immune cells, fibroblasts, bone marrow-derived inflammatory cells, various signaling molecules and extracellular matrix. Macrophages are one of the most important immune cells in TME, which mainly work as recognition, phagocytosis and degradation of foreign bodies, bacteria and dead cells, etc. In addition, macrophages can also play a role in presenting antigens to T cells to initiate adaptive immune response, which means macrophages not only participate in innate immunity, but also adaptive immunity. Macrophages can be divided into M1 and M2 polarization phenotypes, tumor-associated macrophages (TAMs) are a type of macrophages, which status is similar to M2 macrophages [5]. Here, we aimed to explore the role of m6A methylation enzymes ALKBH5 and IGF2BP2 in regulating macrophage polarization in ovarian cancer microenvironment.

M6A methylation is one of the most common RNA modifications, and participates in all stages of RNA life cycle, including RNA transcription, translation and degradation [4]. In fact, m6A methylation also participates in cancer progression. ALKBH5 is one of the most important and classical demethylases. In many cancers, ALKBH5 *functions* as *an oncogene*. In GBM, ALKBH5 worked as an oncogene and influences the self-renewal and proliferation of cancer stem cells [6]. In ovarian cancer, ALKBH5 was up-regulated to enhance the stability of BCL-2, thus inhibiting the autophagy, and promoting the invasion and proliferation of cancer cells [7]. In addition, ALKBH5-HOXA10 loop could promote the cisplatin resistance in epithelial ovarian cancer through demethylating JAK2 [8]. As an important m6A recognition protein, IGF2BP2 mainly acts to promote the stability and translation of mRNA. In ovarian cancer, the knockdown of circ-0001756 could suppress IGF2BP2 mediated RAB5A expression, thus inhibiting malignant progression of ovarian cancer [9]. As for the role that IGF2BP2 plays in immune response, study showed that IGF2BP2 could affect the immune-related biological pathways in oral squamous cell carcinoma, thus leading to the worse prognosis for cancer [10]. However, few studies have focused on the role of ALKBH5 and IGF2BP2 in regulating macrophage polarization in ovarian cancer.

Compared to the M0 macrophages, we first identified that ALKBH5 and IGF2BP2 were up-regulated in M2 macrophages. To deepen our understanding the role of ALKBH5 and IGF2BP2 play in ovarian cancer immune microenvironment, we used various databases to explore their relationships with immune cells, not only at tissues level, but also at single-cell level. Finally, we validated the role of ALKBH5 plays in macrophage polarization through RT-PCR. Taken together, our results could potentially represent the roles of ALKBH5 and IGF2BP2 played in macrophages in ovarian cancer, which might work as the potential immunotherapy biomarkers.

## Materials and methods

### Identification of differentially expressed m6A genes in macrophages

Two independent datasets GSE35495 and GSE36537 from GEO database (https://www.ncbi.nlm.nih.gov/gds/) were included to explore the differentially expressed genes (DEGs) between M0 and M2 macrophages [11, 12]. A venn plot was constructed to explore the co-expressed genes in M2 macrophages in these two datasets. To further validate the relative expression of ALKBH5 and IGF2BP2 in M0 and M2 macrophages, two independent datasets GSE108312 and GSE35449 from GEO database were used.

### Immune analysis of ALKBH5 and IGF2BP2 in ovarian cancer

To evaluate the correlation between ALKBH5 and IGF2BP2 with immune cells in ovarian cancer, TIMER2.0 was used. Online website GSCA (http://bioinfo.life.hust.edu.cn/GSCA) was used to investigate the correlation between mRNA expression and CNV status of ALKBH5 and IGF2BP2 with distinct immune cells in ovarian cancer [13]. Differential expression levels of immune cells in ovarian cancer and normal ovary tissues were investigated in online tool GEPIA2021 (http://gepia2021.cancer-pku.cn) [14]. To investigate the correlation between ALKBH5 and M2 macrophage markers IL-10 and CD163 in the TME of ovarian cancer, dataset GSE158739 was used, which contains data on tumor-associated macrophages derived from TME of ovarian cancer. To further prove the correlation between ALKBH5 and IGF2BP2 with M2 macrophages based on CIBERSORT method in ovarian cancer, two independent ovarian cancer datasets GSE44104 and GSE65986 from GEO database were used.

### Prognostic value of ALKBH5 and IGF2BP2 in ovarian cancer

To evaluate the prognostic role of ALKBH5 and IGF2BP2 played in ovarian cancer, overall survival (OS) analysis and progression-free survival (PFS) analysis were conducted using the online tools K-M plotter (https://kmplot.com/analysis) and TIMER2.0 (http://timer.cistrome.org) [15, 16]. The expressions of ALKBH5 and IGF2BP2 in cell localization were explored in online website The Human Protein Atlas (HPA) software (https://www.proteinatlas.org) [17]. The small molecules or drugs targeting ALKBH5 and IGF2BP2 were investigated in GSDC and CTRP databases.

### Mutation analysis of ALKBH5 and IGF2BP2 in ovarian cancer

The copy-number alterations and gene mutations of ALKBH5 and IGF2BP2 in ovarian cancer were investigated in cBioPortal database. To explore the CNV status of genes and their mRNA expressions, GSCA database was used. The most correlation genes related to ALKBH5 and IGF2BP2 were investigated in cBioPortal database.

## Single-cell analysis of ALKBH5 and IGF2BP2 in ovary and ovarian cancer tissues

The single-cell analysis of ALKBH5 and IGF2BP2 expression in immune cells was investigated in online sc-TIME Portal software (http://sctime.sklehabc.com/unicellular/home) in ovarian cancer [18]. Furthermore, we explored ALKBH5 and IGF2BP2 expressions and their correlation with various types of immune cells in ovarian tissues in HPA.

### Cell culture and treatment

The human monocyte cell line THP-1 was purchased from Beijing Beina Chuanglian Biotechnology Institute. Cells were cultured in RPMI1640 with 10% FBS with 5% CO2 at 37℃. In order to induce macrophages, THP-1 was treated with 100 ng/ml PMA for 24 h. To manipulate the inhibition and overexpression of ALKBH5 in macrophages, we constructed lentivirus that could knockdown and overexpress ALKBH5 from Shanghai GenePharma Biotechnology Co., Ltd.

### RNA extraction and RT-PCR

Total RNA was extracted using Trizol reagent. To reverse transcribe mRNA into cDNA, we used the PrimeScript RT reagent Kit with gDNA Eraser. SYBR Premix Ex Taq was used to perform quantitative RT-PCR through ABI 7500 Fast instrument. The sequences of the primers were shown in Supplementary Table 1. GAPDH was used as the internal reference.

## Functional analysis of ALKBH5 in ovarian cancer

Ovarian cancer patients from TCGA dataset were divided into two groups based on ALKBH5 expression level. The DEGs between these two groups were investigated through R software using “limma” package, genes were collected with *p*-value < 0.05 and Fold Change > 1.2. Online website STRING (https://cn.string-db.org) was used to construct the PPI network [19]. Cytoscape was used to identify the hub genes. Online website DAVID (https://david.ncifcrf.gov) was used to perform GO and KEGG analysis based on DEGs [20].

### Statistical analysis

Correlations between ALKBH5 and IGF2BP2 with immune-related genes and other m6A methylation enzymes were explored based on TCGA dataset and GEO dataset using Pearson method. The correlation between ALKBH5 and IGF2BP2 with M2 macrophage markers IL-10 and MRC1 were explored in GEPIA database. All tests were evaluated with *p*-value < 0.05. GraphPad Prism 7 software was used to analyze all the data. Differences between two groups were tested using the Student’s t-test or Chi-square test.

## Results

### ALKBH5 and IGF2BP2 were up-regulated in M2 macrophages

**Fig. 1:**
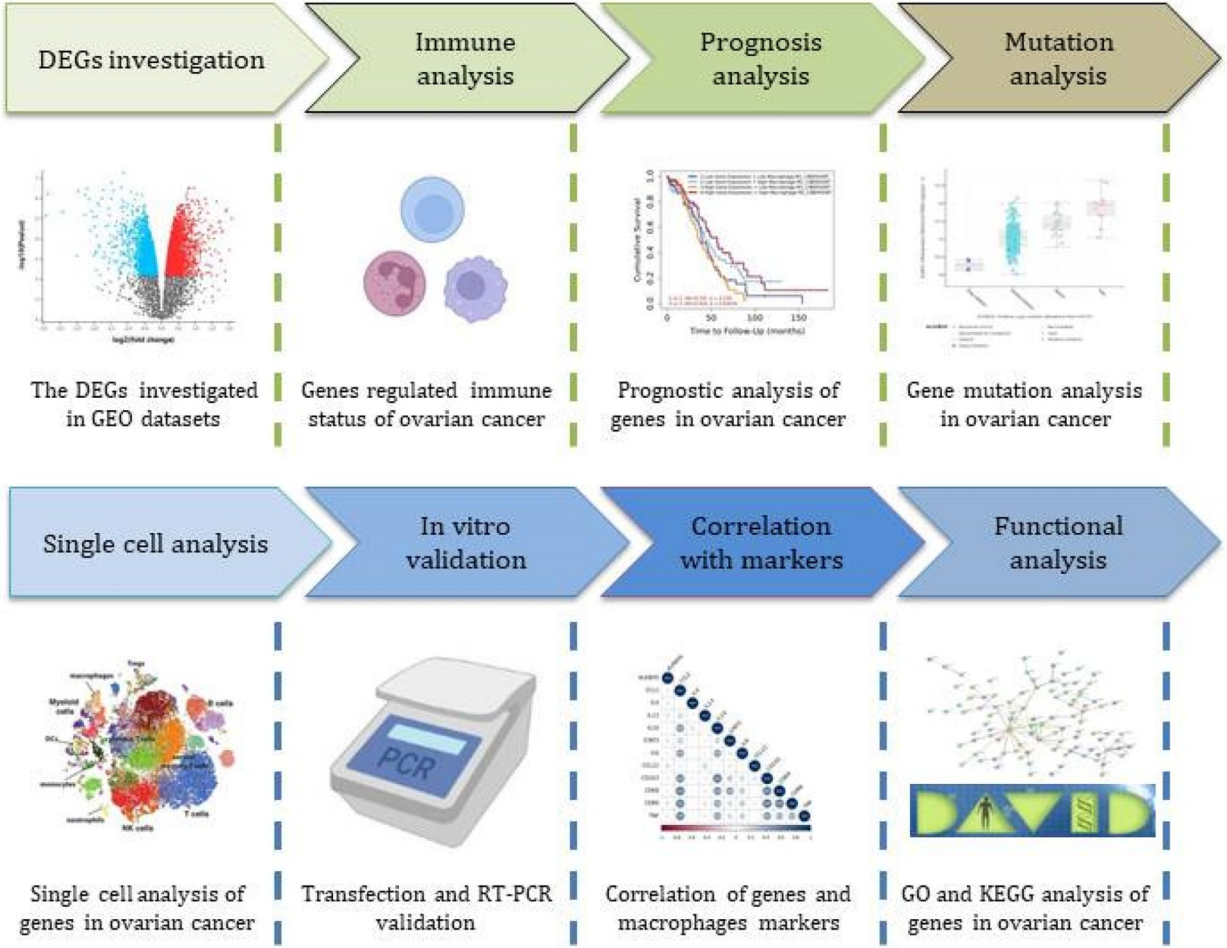
Flow diagram of the study design

**Fig. 2:**
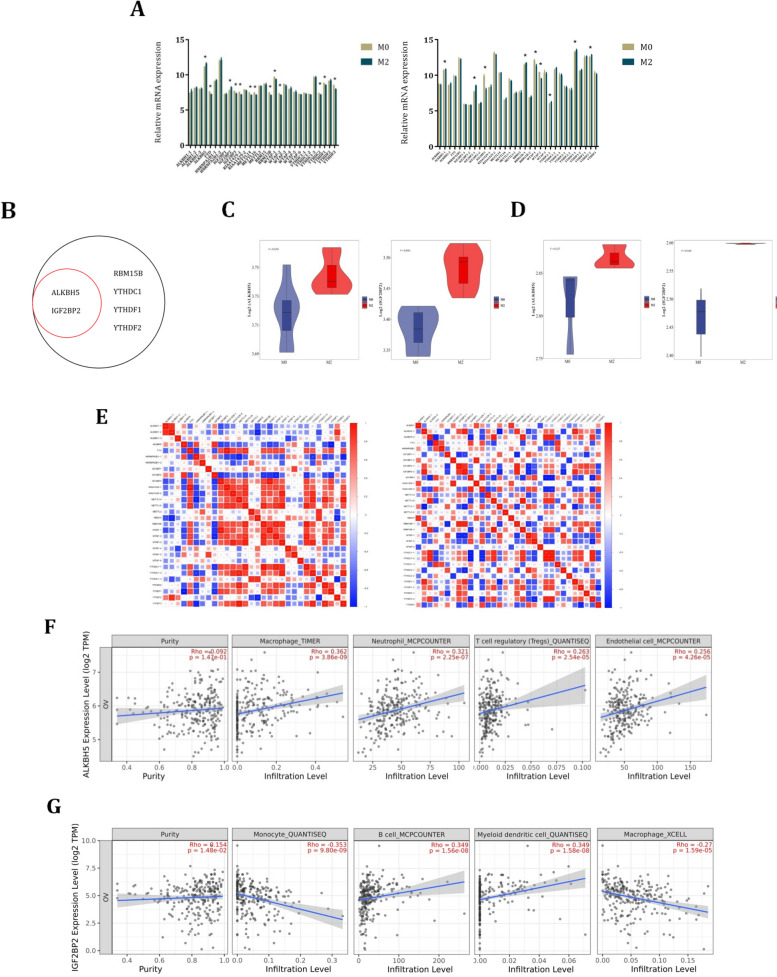
ALKBH5 and IGF2BP2 were up-regulated in M2 macrophages. **A** The differential expression of m6A methylation enzymes between M0 and M2 macrophages in GSE35495 and GSE36537, the left plot represented macrophage samples from GSE35495 with three M0 macrophage samples and three M2 macrophage samples, the right plot represented macrophage samples from GSE36537 with three M0 macrophage samples and three M2 macrophage samples. **B** Venn diagram to analyze co-expression genes in GSE35495 and GSE36537. **C** The relative expression of ALKBH5 and IGF2BP2 in M0 and M2 macrophages in GSE108312 with three M0 macrophage samples and three M2 macrophage samples. **D** The relative expression of ALKBH5 and IGF2BP2 in M0 and M2 macrophages in GSE35449 with seven M0 macrophage samples and seven M2 macrophage samples. **E** The correlation between m6A methylation enzymes in macrophages in GSE35495 and GSE36537. **F** The correlation between expression of ALKBH5 and different immune cells in ovarian cancer using TIMER2.0. **G** The correlation between expression of IGF2BP2 and different immune cells in ovarian cancer using TIMER2.0

In Fig. 1, we constructed a flow diagram to represent our study design. In this study, we collected two independent GEO datasets, GSE35495 and GSE36537 to demonstrate the DEGs between M0 and M2 macrophages (Supplementary Fig. 1A-B). All the m6A methylation enzymes were *analyzed* to *demonstrate differential* expression in M0 and M2 macrophages, which showed most of the enzymes exhibited distinct expression between these two types of macrophages (Fig. 2A). However, the m6A methylation enzymes which were up-regulated in M2 macrophages were not that much. In the venn diagram, we found that only ALKBH5 and IGF2BP2 worked as the genes that up-regulated in M2 macrophages both in GSE36537 and GSE39495 (Fig. 2B). To further validate the relative expression of ALKBH5 and IGF2BP2 in M0 and M2 macrophages, GEO datasets GSE108312 and GSE35449 were used. The results showed that ALKBH5 and IGF2BP2 were both expressed highly in M2 macrophages (Fig. 2C-D). In Fig. 2E, we investigated the correlation between the m6A methylation enzymes in the two GEO datasets, which showed most of the enzymes had closely correlation. Heatmap plot was used to represent the m6A methylation enzymes in these two datasets (Supplementary Fig. 1C). We found that the expression of ALKBH5 was relatively high in macrophages, while IGF2BP2 was expressed at low levels in macrophages. Next, we would like to investigate the correlation of ALKBH5 and IGF2BP2 with immune cells expression in ovarian cancer microenvironment. Thus, we further investigated the role of ALKBH5 and IGF2BP2 played in immune cells using TIMER2.0 database. Results showed that the expression of ALKBH5 significantly correlated with macrophage, neutrophil, Tregs and endothelial cells expression, especially in macrophages had the closest correlation with R = 0.362 (Fig. 2F). In IGF2BP2 showed close correlation with monocytes, B cells, myeloid dendritic cells and macrophages, while the IGF2BP2 showed the closest correlation with monocytes with R = -0.353 (Fig. 2G). These results suggested that ALKBH5 and IGF2BP2 showed closely correlation with macrophage expression in ovarian cancer, which might participate in regulating the polarization of macrophages in ovarian cancer microenvironment.

### ALKBH5 and IGF2BP2 correlated with the expression of immune cells in ovarian cancer

**Fig. 3:**
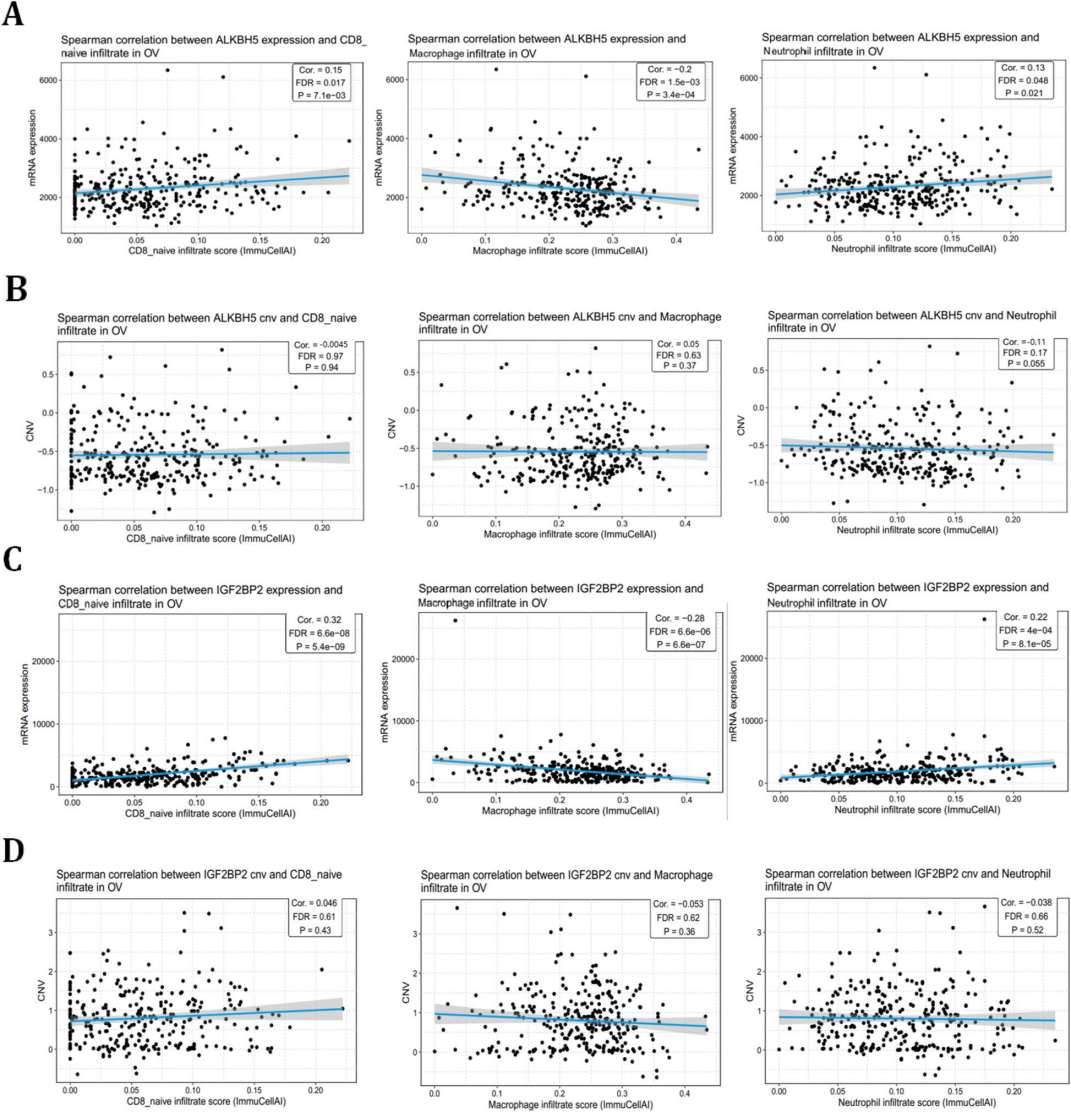
ALKBH5 and IGF2BP2 correlated with immune cells in ovarian cancer. **A** The correlation between mRNA expression of ALKBH5 and different immune cells in ovarian cancer using GSCA. **B** The correlation between ALKBH5 CNV mutation and different immune cells in ovarian cancer using GSCA. **C** The correlation between mRNA expression of IGF2BP2 and different immune cells in ovarian cancer using GSCA. **D** The correlation between IGF2BP2 CNV mutation and different immune cells in ovarian cancer using GSCA

Further validated the roles of ALKBH5 and IGF2BP2 played in ovarian cancer, GSCA software were used. Results showed that ALKBH5 and IGF2BP2 mRNA levels correlated with CD8 naïve cells, macrophages and neutrophils expressions (Fig. 3A & C). However, there was no significant relationship between the CNV mutation of ALKBH5 and IGF2BP2 with the expression of those immune cells in ovarian cancer (Fig. 3B & D). Based on these analysis, we found that ALKBH5 and IGF2BP2 could regulate the immune status of ovarian cancer microenvironment mainly through its mRNA expression levels, the mutation status of ALKBH5 and IGF2BP2 might not influence the immune status of ovarian cancer. These results represented that ALKBH5 and IGF2BP2 may be involved in regulating the immune status based on their expression value in immune cells, especially in macrophages.

### Overexpression of ALKBH5 and IGF2BP2 correlated with worse prognosis in ovarian cancer

**Fig. 4:**
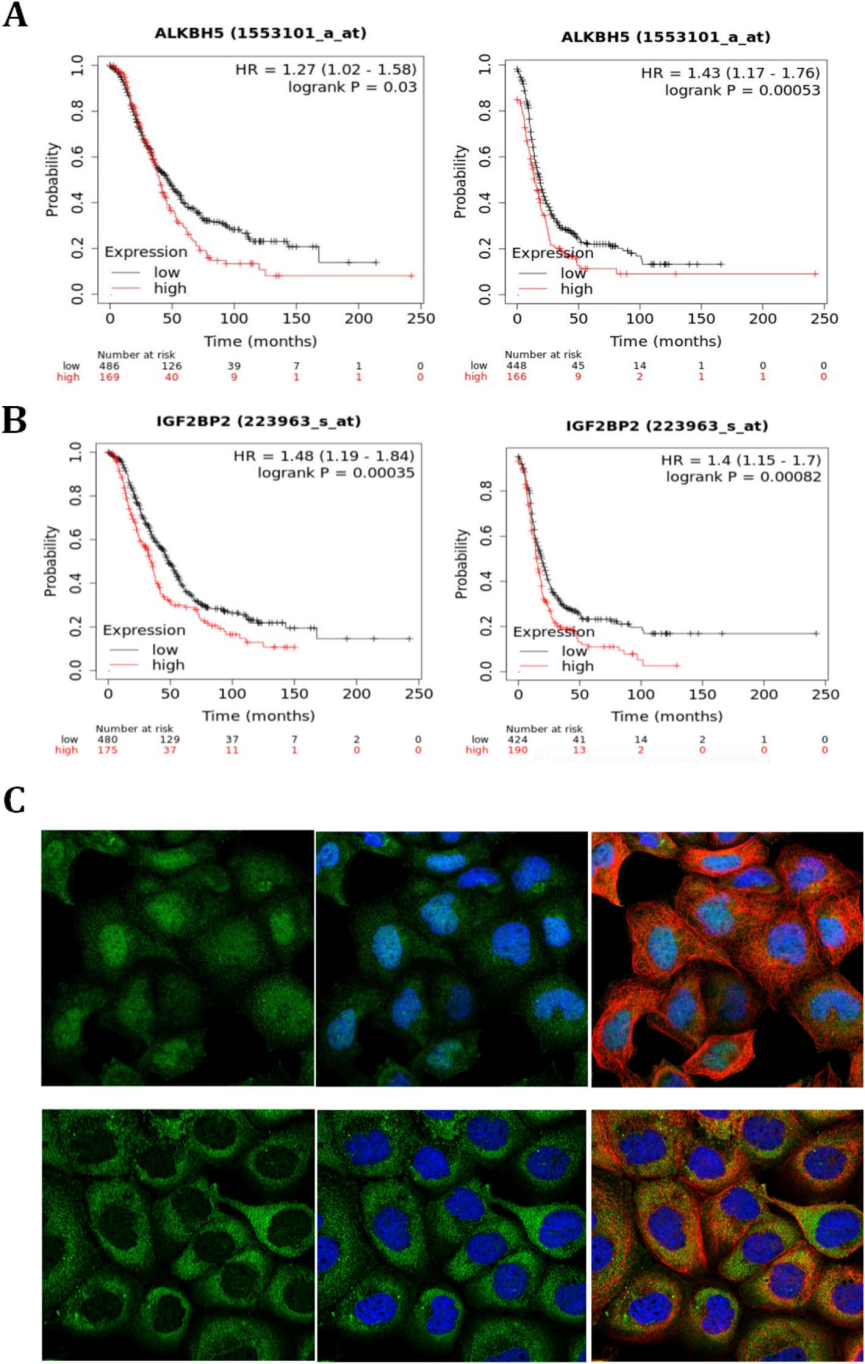
ALKBH5 and IGF2BP2 correlated with the prognosis of ovarian cancer. **A** OS and PFS analysis of ALKBH5 in ovarian cancer from K-M plotter. **B** OS and PFS analysis of IGF2BP2 in ovarian cancer from K-M plotter. **C** The localization of ALKBH5 and IGF2BP2 in A-431 cell line. Green color represented target protein, blue color represented cell nucleus, and red color represented microtubulues. Therefore, the upper left figure represented ALKBH5 expression, the upper middle figure represented colocalization of ALKBH5 and cell nucleus, the upper right figure represented colocalization of ALKBH5, cell nucleus and microtubulues. The lower left figure represented IGF2BP2 expression, the lower middle figure represented colocalization of IGF2BP2 and cell nucleus, the lower right figure represented colocalization of IGF2BP2, cell nucleus and microtubulues

To investigate the prognostic role of ALKBH5 and IGF2BP2 played in ovarian cancer, we used the online K-M plotter database. The results showed that high expression of ALKBH5 and IGF2BP2 was associated with a poorer prognosis both in OS and PFS analysis in ovarian cancer, which might be due to its high expression in M2 macrophages in ovarian cancer microenvironment (Fig. 4A-B). In cell localization, ALKBH5 was widely expressed in the nucleus and cytoplasm of cells, with a predominant expression in the nucleus, while IGF2BP2 was mainly expressed in cytoplasm of the cells (Fig. 4C).

### ALKBH5 correlated with M2 macrophages markers in ovarian cancer

**Fig. 5:**
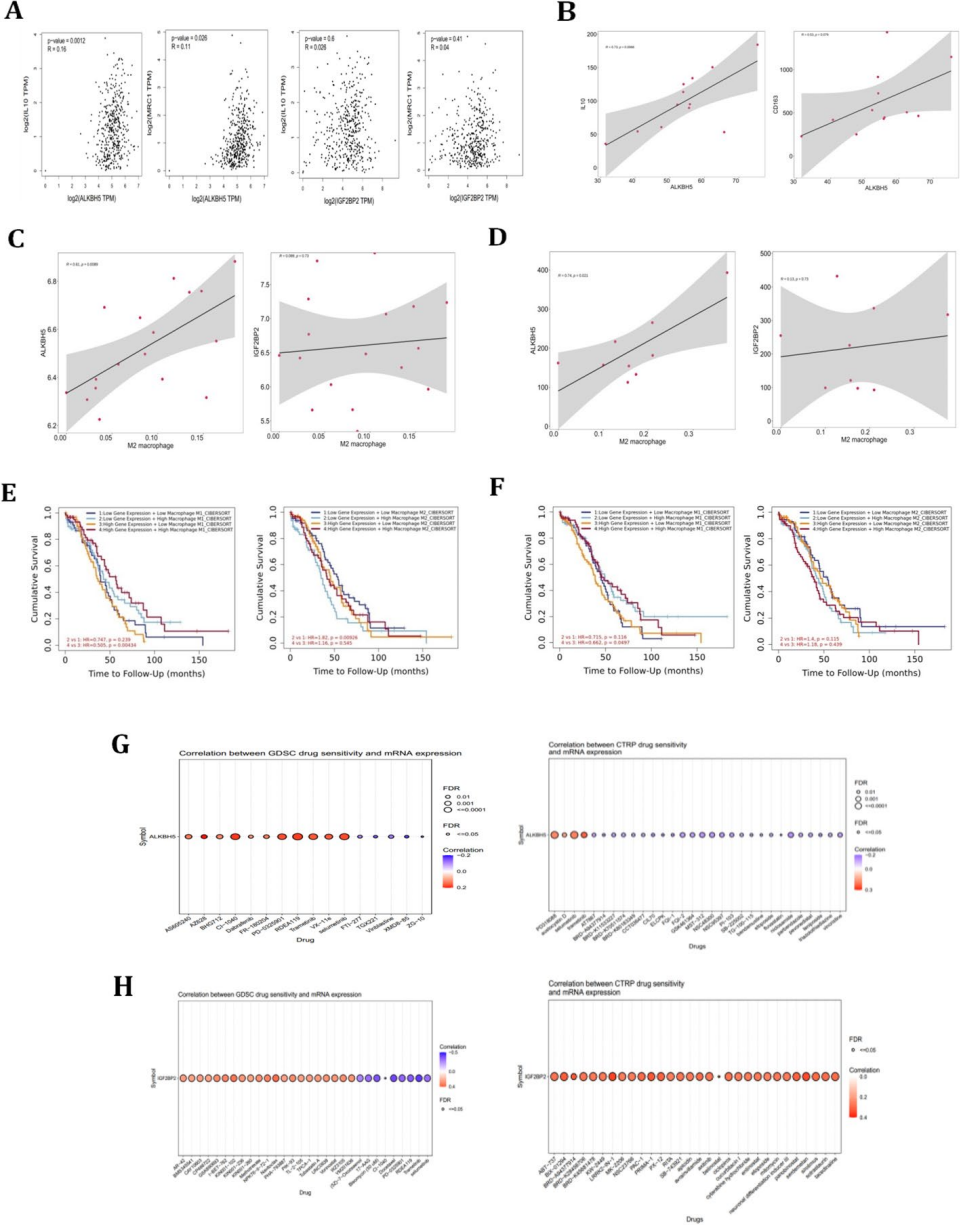
ALKBH5 correlated with M2 macrophage markers in ovarian cancer. **A** The correlation between ALKBH5 and IGF2BP2 with M2 polarization markers MRC1 and IL-10 in ovarian cancer. **B** The correlation between ALKBH5 and M2 macrophage markers IL-10 and CD163 in macrophages derived from TME of ovarian cancer in GSE158739. **C** The correlation between ALKBH5 and IGF2BP2 with M2 macrophages in serous ovarian cancer through GSE44104. **D** The correlation between ALKBH5 and IGF2BP2 with M2 macrophages in serous ovarian cancer through GSE65986. **E** Combined OS analysis of ALKHB5 and expression of M1 and M2 macrophages in ovarian cancer from TIMER2.0. **F** Combined OS analysis of IGF2BP2 and expression of M1 and M2 macrophages in ovarian cancer from TIMER2.0. **G** The small molecules or drugs targeting ALKBH5 in ovarian cancer. **H** The small molecules or drugs targeting IGF2BP2 in ovarian cancer

Furthermore, we tried to investigate the role of ALKBH5 and IGF2BP2 played in macrophages in ovarian cancer. In order to investigate whether ALKBH5 and IGF2BP2 regulated the immune status of ovarian cancer by influencing the polarization of macrophages, we investigated that the correlation between ALKBH5 and IGF2BP2 with M2 macrophage polarization-related genes. In Fig. 5A, we found that ALKBH5 positively correlated with the expression of M2 macrophages markers IL-10 (*p*-value = 0.0012) and MRC1 (*p*-value = 0.026), which indicated ALKBH5 might participate in promoting M2 macrophages polarization. However, there was no significant correlation between IGF2BP2 with the polarization markers IL-10 and MRC1. To further prove the relationship between ALKBH5 and M2 macrophage markers in TME of ovarian cancer, we found that ALKBH5 positively correlated with M2 macrophage markers IL-10 and CD163 through Pearson analysis in ovarian cancer related dataset GSE158739 (Fig. 5B). In addition, to investigate the relationship between M2 macrophages with ALKBH5 and IGF2BP2 in ovarian cancer, two independent datasets from GEO database using CIBERSORT method were searched for further study. In both GSE44104 and GSE65986, the results showed that M2 macrophages positively correlated with ALKBH5 in serous ovarian cancer, while no significant correlation between M2 macrophages and IGF2BP2 was found (Fig. 5C-D). Thus, we assumed that ALKBH5, but not IGF2BP2 might participate in regulation the macrophages in ovarian cancer, especially by promoting the M2 polarization of macrophages. Furthermore, we analyzed the prognostic value of ovarian cancer based on the expression level of ALKBH5 and IGF2BP2 with distinct phenotypes of macrophage expressions (Fig. 5E-F). The results showed that in M1 macrophage group, patients with high expression of ALKBH5 and low expression of M1 macrophages had the worst prognosis. While in M2 macrophage group, patients with low expression of ALKBH5 and low level of M2 macrophages exhibited the best prognosis. However, no obvious survival differences were observed in IGF2BP2 combined analysis with macrophages. The small molecules or drugs targeting ALKBH5 or IGF2BP2 were investigated using GDSC and CTRP database, which might help demonstrate the potential drugs that target ALKBH5 or IGF2BP2 in ovarian cancer (Fig. 5G-H).

### The mutation analysis of ALKBH5 and IGF2BP2 in ovarian cancer

**Fig. 6:**
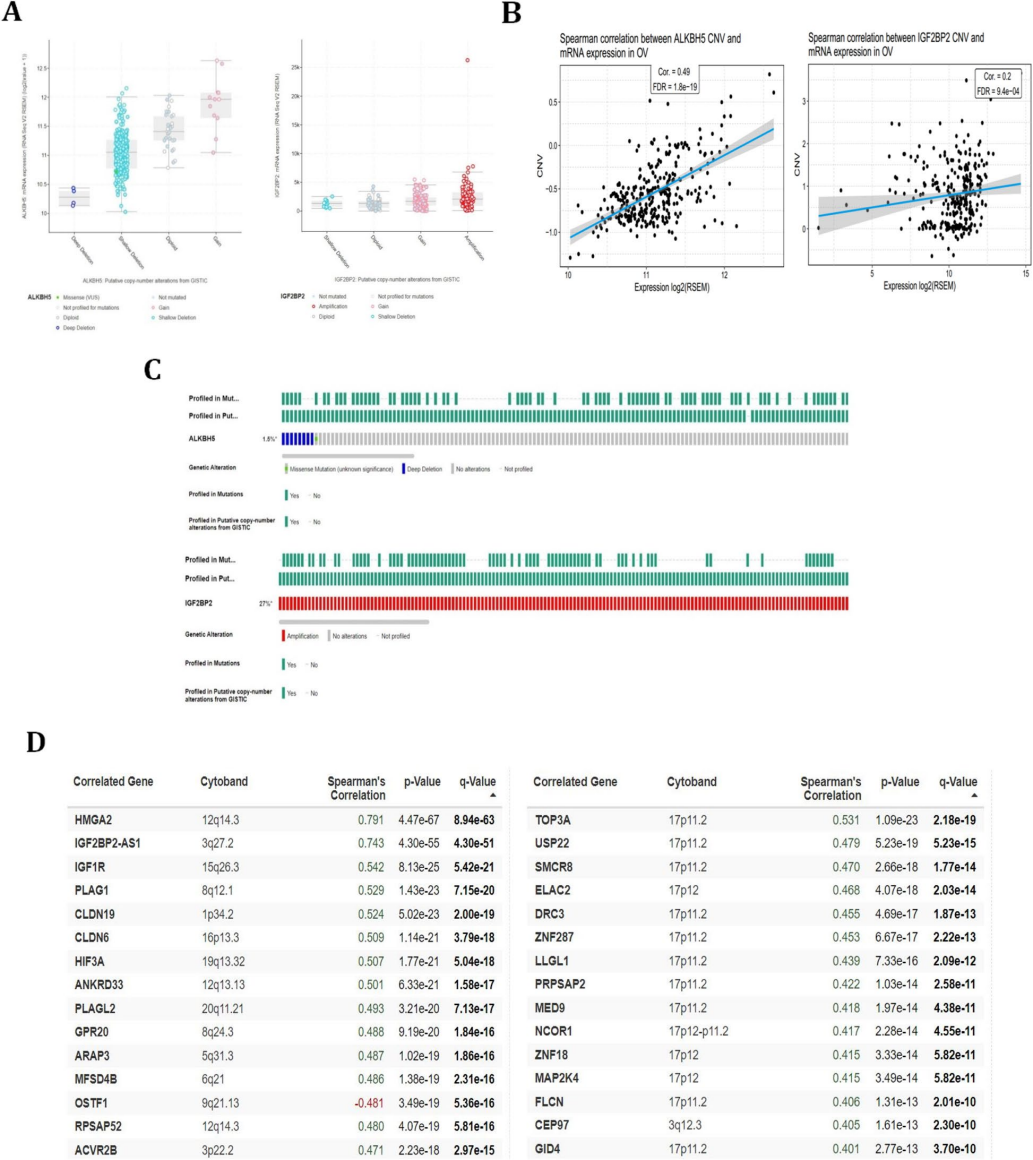
The mutation status of ALKBH5 and IGF2BP2 in ovarian cancer. **A** The copy number alteration of ALKBH5 and IGF2BP2 in ovarian cancer. **B** The correlation between copy number alteration of ALKBH5 and IGF2BP2 with mRNA expression. **C** The mutation status of ALKBH5 and IGF2BP2 in ovarian cancer. **D** The 15 top genes correlated with ALKBH5 and IGF2BP2 in ovarian cancer. The left figure represented ALKBH5, and the right figure represented IGF2BP2

In cBioPortal database, we analyzed the copy number alterations of ALKBH5 and IGF2BP2 in ovarian cancer. The copy-number alterations of ALKBH5 and IGF2BP2 in ovarian cancer were shown in Fig. 6A. The results showed that distinct copy-number alteration of ALKBH5 represented different levels of ALKBH5 mRNA. However, no obvious mRNA level of IGF2BP2 was observed in different copy-number alteration of IGF2BP2. Similarly, in GSCA database, we analyzed the correlation between CNV status of ALKBH5 and IGF2BP2 with mRNA levels. We found that ALKBH5 mRNA level showed a significant correlation with CNV status, however, IGF2BP2 didn’t show the obvious correlation (Fig. 6B). It is worth noting that we described the correlation between ALKBH5 or IGF2BP2 CNV status and immune cells infiltration in Fig. 3. Different from Fig. 3, we described the correlation between ALKBH5 CNV status with its mRNA expression levels in Fig. 6. In total, we found that mutation of ALKBH5 is only 1.5% in ovarian cancer, however, the mutation of IGF2BP2 accounts for 27% of ovarian cancer patients (Fig. 6C). Finally, the top 15 genes correlated with ALKBH5 and IGF2BP2 in ovarian cancer were shown in Fig. 6D.

### The single-cell analysis of ALKBH5 and IGF2BP2 in immune cells in ovarian cancer

**Fig. 7:**
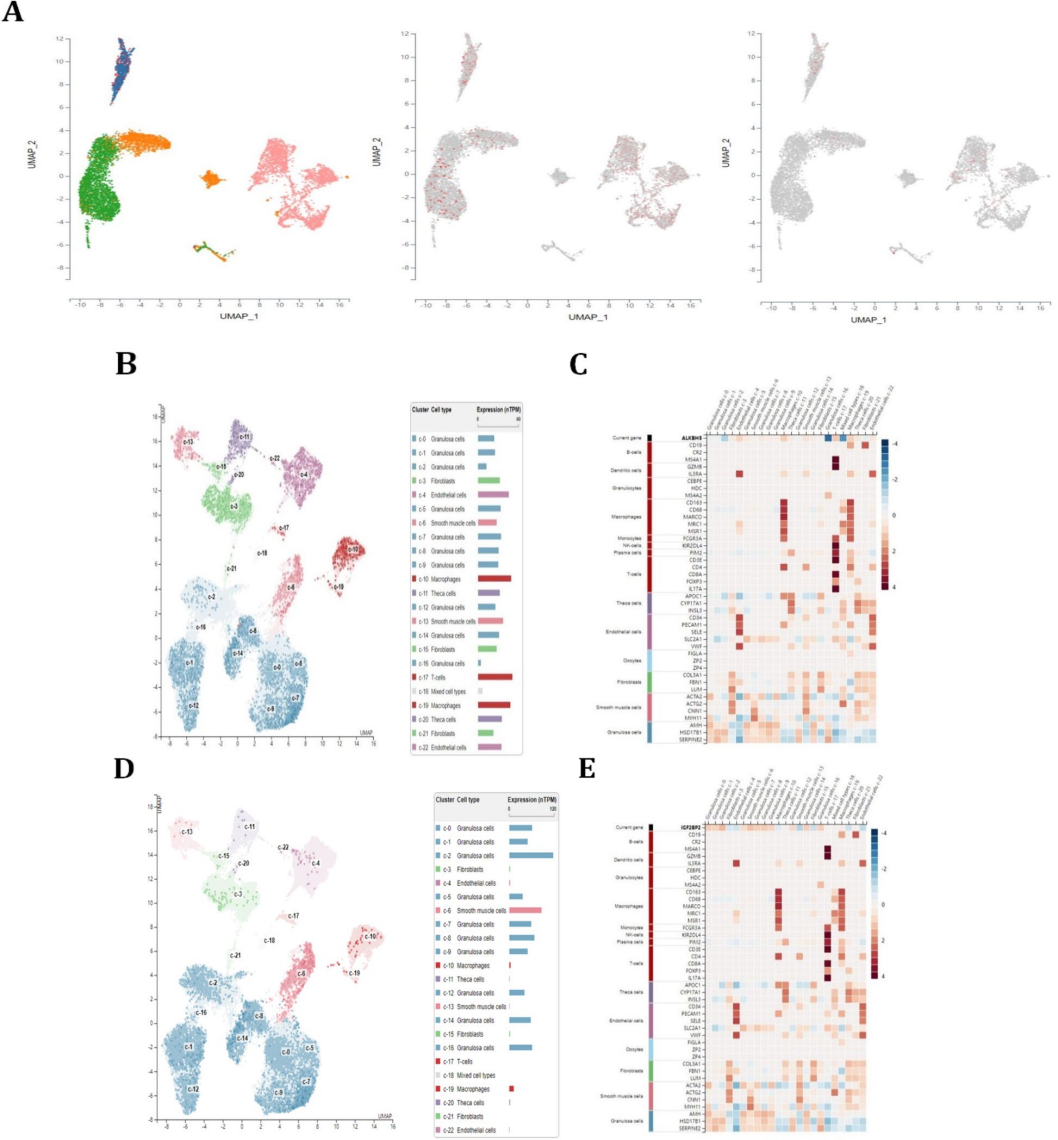
Single-cell analysis of ALKBH5 and IGF2BP2 in ovary and ovarian cancer tissues. **A** The expression of ALKBH5 and IGF2BP2 in distinct immune cells in ovarian cancer microenvironment using sc-TIME. The orange one represented ascites DCs, the green one represented ascites macrophages, the red one and the blue one represented the monocytes, while the pink one represented tonsil DCs. The middle one represented ALKBH5, while the right one represented IGF2BP2. **B** Single-cell analysis of ALKBH5 in distinct immune cells in ovary tissues. **C** Correlation between ALKBH5 and distinct immune cells markers in ovary tissues. **D** Single-cell analysis of IGF2BP2 in distinct immune cells in ovary tissues. **E** Correlation between IGF2BP2 and distinct immune cells markers in ovary tissues

To explore the role of ALKBH5 and IGF2BP2 in immune cells at single-cell level in ovarian cancer, we found that ALKBH5 was widely expressed in immune cells, but its expression was highly expressed in macrophages (Fig. 7A). However, the expression of IGF2BP2 was very low in immune cells in ovarian cancer microenvironment. In the single-cell analysis of ovary tissues, we found that ALKBH5 was expressed highest in the blood and immune cells (Fig. 7B). Further investigated the expression of ALKBH5 in immune cells in detail, we found ALKBH5 was expressed at the highest levels in macrophages and T cells, especially in macrophages, which showed positive correlation with the macrophages markers CD163, CD68, MARCO, MRC1 and MSR1 (Fig. 7C). These results suggested that ALKBH5 also showed significantly correlation with immune cell, especially macrophages at the single-cell level in ovarian cancer. However, we found that the expression of IGF2BP2 was mainly expressed in granulosa cells and smooth muscle cells, its expression in the macrophages of *ovarian* tissues and ovarian cancer tissues in single-cell level analysis were relative very low (Fig. 7D-E). What’s more, in Supplementary Fig. 2A, we also found the expression of ALKBH5 was relatively high in most tissues, especially in ovarian tissues. However, the expression of IGF2BP2 was very low in most of the tissues, including ovarian tissues. The expression of ALKBH5 in distinct subtypes of ovarian cancer cells was also much higher than IGF2BP2, including differentiated, immunoreactive, mesenchymal and proliferative ovarian cancer (Supplementary Fig. 2B). Thus, we paid more attention to investigating the role that ALKBH5 played in ovarian cancer.

### ALKBH5 promoted the M2 polarization of macrophages in ovarian cancer

**Fig. 8:**
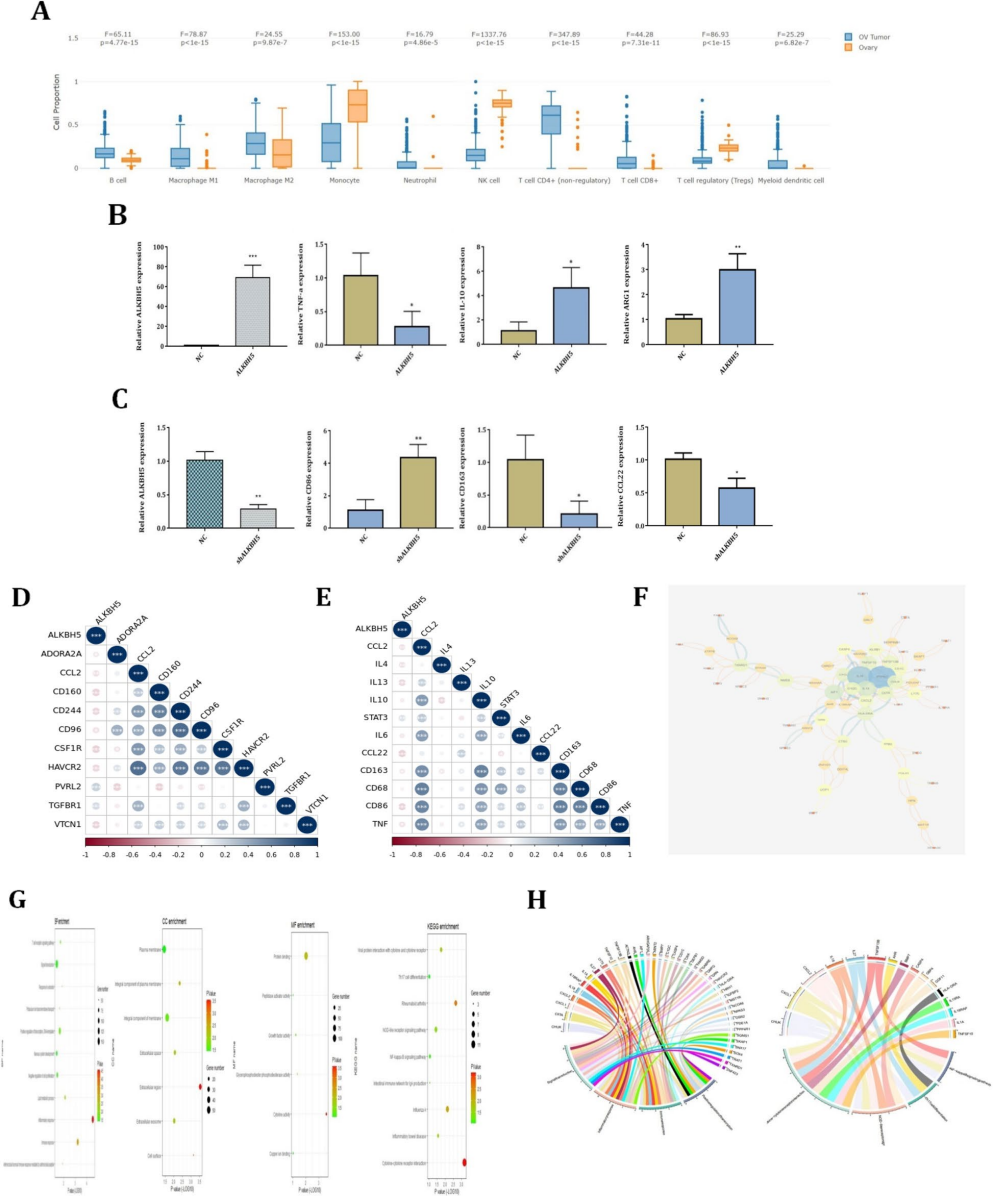
ALKHB5 promoted M2 macrophage polarization in ovarian cancer. **A** Differential expression of ALKBH5 in normal ovary and ovarian cancer immune cells. **B** Overexpression of ALKBH5 in macrophages inhibited TNF-α expression and promoted IL-10, ARG-1 expression. ***C*** Inhibition of ALKBH5 in macrophages promoted CD86 expression and inhibited CD163, CCL22 expression. **D** Correlation between ALBKH5 and immune inhibitors in ovarian cancer. **E** Correlation between ALBKH5 and macrophages polarization markers in ovarian cancer. **F** PPI network constructed according to the DEGs of ALKBH5 in ovarian cancer using cytoscape. **G** Bubble plots showed GO and KEGG analysis of DEGs based on TCGA. **H** Circos plots showed the GO and KEGG analysis of DEGs based on TCGA

To investigate the distinct roles of ALKBH5 played in ovarian cancer and normal ovary immune microenvironment. In Fig. 8A, we found that the immune cells expressions in these two immune microenvironments showed significant differences. Most importantly, the expression of M1 and M2 macrophages were significantly higher expressed in ovarian cancer. However, the expressions of monocytes were significantly higher expressed in normal ovary tissues. We used PMA to differentiate THP-1 cells into macrophages and then we transfected lentivirus into macrophages, which could overexpress or inhibit the expression of ALKBH5 in macrophages. *In *Fig. 8B, when we overexpressed ALKBH5 in macrophages, the results showed that down-regulation of M1 macrophage marker TNF-α, up-regulation of M2 macrophage marker IL-10 and ARG-1. In Fig. 8C, when we inhibited the expression of ALKBH5 in macrophages, the results showed that up-regulation of M1 macrophage marker CD86, down-regulation of M2 macrophage marker CD163 and CCL22. These results indicated that ALKBH5 could promote the M2 polarization of macrophages. Thus, we explored the correlation between ALKBH5 and immune inhibitors, which also showed ALKBH5 might influence the immune response in the ovarian cancer immune microenvironment (Fig. 8D). Finally, the correlation between ALKBH5 and macrophage polarization markers in ovarian cancer was investigated using the Pearson method, as shown in Fig. 8E. Results showed that ALKBH5 correlated with most of the macrophage markers, which meant that ALKBH5 closely correlation with macrophages polarization. Due to the expression value of ALKBH5, ovarian cancer patients were divided into two groups. The DEGs were collected to investigate the biological role of ALKBH5 played in ovarian cancer (Supplementary Table 2). A PPI network was constructed through STRING software and Cytoscape based on DEGs (Fig. 8F). Top 20 hub genes were identified using cytoHubba (Suppelementary Fig. 3A). All the DEGs were collected to investigate GO and KEGG analysis (Table 1). Results represented that ALKBH5 was associated with immune response and inflammatory response in ovarian cancer (Fig. 8G-H). The pathways that ALKBH5 correlated in ovarian cancer were also immune-related pathways, such as Th17 cell differentiation, NOD-like receptor signaling pathway and NF-κB signaling pathway (Table 2). In Supplementary Fig. 2B-C, we found that ALKBH5, but not IGF2BP2 correlated with the apoptosis pathways and MAPK pathways in ovarian cancer.

**Table 1:** GO analysis of DEGs according to the expression of ALKBH5 in ovarian cancer

Ontology	ID	Description	Count	*P-*value	Fold enrichment
BP	GO: 0006954	Inflammatory response	14	0.0000296	4.201085608
BP	GO:0006955	Immune response	13	0.000604	3.288925209
BP	GO:0006629	Lipid metabolic process	7	0.007533531	4.069801683
BP	GO:0061844	Antimicrobial humoral immune response mediated by antimicrobial peptide	5	0.012541582	5.547305046
BP	GO:0032355	Response to estradiol	5	0.013740931	5.398716518
BP	GO:0050852	T cell receptor signaling pathway	6	0.018869267	3.901008065
BP	GO:0045893	Positive regulation of transcription, DNA-templated	12	0.022040078	2.175674663
BP	GO:0071805	Potassium ion transmembrane transport	5	0.022460029	4.651201923
BP	GO:0008285	Negative regulation of cell proliferation	9	0.035365282	2.381578228
BP	GO:0007165	Signal transduction	17	0.039221453	1.707501038
BP	GO:0007399	Nervous system development	8	0.040573389	2.50634715
CC	GO:0005576	Extracellular region	34	0.000291	1.916231801
CC	GO:0009986	Cell surface	15	0.000699	2.891282943
CC	GO:0005887	Integral component of plasma membrane	23	0.004477562	1.896484463
CC	GO:0005615	Extracellular space	27	0.008560599	1.685749584
CC	GO:0070062	Extracellular exosome	30	0.009312576	1.614527868
CC	GO:0016021	Integral component of membrane	58	0.024020882	1.281279272
CC	GO:0005886	Plasma membrane	53	0.035827021	1.275180742
MF	GO:0005125	Cytokine activity	9	0.000259	5.421373556
MF	GO:0005515	Protein binding	124	0.009391396	1.133723536
MF	GO:0008889	Glycerophosphodiester phosphodiesterase activity	2	0.04267721	45.7804878
MF	GO:0008083	Growth factor activity	5	0.056972199	3.447325889
MF	GO:0016504	Peptidase activator activity	2	0.091506587	20.80931264
MF	GO:0005507	Copper ion binding	3	0.09307753	5.819553535

**Table 2:** KEGG analysis of DEGs according to the expression of ALKBH5 in ovarian cancer

Ontology	ID	Description	Count	P-value	Fold enrichment
KEGG	Hsa04060	Cytokine-cytokine receptor interaction	11	0.000662	3.688797024
KEGG	Hsa05323	Rheumatoid arthritis	6	0.002293243	6.382376082
KEGG	Hsa05164	Influenza A	7	0.007076995	4.049636286
KEGG	Hsa04061	Viral protein interaction with cytokine and cytokine receptor	5	0.017545843	4.946341463
KEGG	Hsa05321	Inflammatory bowel disease	4	0.02689861	6.087804878
KEGG	Hsa04621	NOD-like receptor signaling pathway	6	0.036491698	3.225874867
KEGG	Hsa04064	NF-kappa B signaling pathway	4	0.085387502	3.804878049
KEGG	Hsa04672	Intestinal immune network for IgA production	3	0.085719612	6.056744649
KEGG	Hsa04659	Th17 cell differentiation	4	0.09311625	3.66395664

## Discussion

Ovarian cancer is always characterized by the late stage once found, with high recurrence rate and mortality. It is known as the “silent killer” for women. Studies have shown that more than 25% of ovarian cancer patients represented chemoresistance at the first relapse [21]. So it is important to find new approaches for ovarian cancer therapy. M6A methylation is the most common nucleotide modification in mRNA, about 1/4 mRNA molecules contain at least one m6A modification site [22]. M6A modification is involved in various processes of mRNA metabolism [23–25]. Recent studies have shown that m6A could affect a variety of cellular biological processes and played an important role in cell fate determination, lipid metabolism and immunity [26–28]. ALKBH5 works as an important demethylase, while IGF2BP2 works as the m6A recognition protein. However, its role in ovarian cancer needs further study. We first found that ALKBH5 and IGF2BP2 were up-regulated in M2 macrophages, and both showed significantly correlated with immune cells in ovarian cancer, especially macrophages. Importantly, we found that the closely correlation between ALKBH5 and IGF2BP2 with immune cells due to its mRNA expression levels, rather than their CNV status. Furthermore, we investigated the prognostic role of ALKBH5 and IGF2BP2 in ovarian cancer, which both showed the overexpression of ALKBH5 and IGF2BP2 represented the worse prognosis for ovarian cancer.

The mechanisms that can regulate the macrophages polarization has been investigated for years. However, the studies focused on investigating the role that m6A methylation-related enzymes that played in cancer immune microenvironment were very limited. According to our analysis, we found that ALKBH5 and IGF2BP2 were significantly correlated with the immune response in ovarian cancer. ALKBH5 is a classical m6A demethylase that primarily functions to remove m6A modification to promote mRNA nuclear processing and export. In ovarian cancer, high expression of TLR4 could activate NF-κB signaling pathway, thus up-regulated ALKBH5 to influence the m6A methylation of NANOG, which further promoted carcinogenesis in ovarian cancer [29]. In epithelial ovarian cancer, ALKBH5-HOXA10 loop regulated the methylation of JAK2, which could activate JAK2-STAT3 signaling, thus promoting chemoresistance of cancer cells [8]. Moreover, IGF2BP2 worked as an important m6A recognition protein, which mainly acted to promote the stability and translation of mRNA. In ovarian cancer, the study showed that circITGB6 can directly interact with IGF2BP2 and FGF9, thus stabilized FGF9 and promoted M2 polarization in ovarian cancer [30]. In fact, research on whether the expression of ALKBH5 and IGF2BP2 can regulate the polarization of macrophages were very limited. Most of the studies correlating m6A methylation enzymes and macrophages function have focused on studying the role of METTL3 in macrophages. In myeloid cells, the ablation of METTL3 could promote tumor metastasis, which was due to the deficiency of METTL3 increased the M1 macrophages and regulatory T cells infiltration [31]. In breast cancer, METTL14 and ZC3H13 were down-regulated, indicating a positive correlation between the expression of METTL14 and ZC3H13 positively correlated with various kinds of immune cells, including macrophages, T cells and DCs [32]. In this study, we found that among all the immune cells, ALKBH5 and IGF2BP2 showed the closest correlation with macrophages in ovarian cancer. However, we found that the expression level of IGF2BP2 was much lower than ALKBH5 in ovarian cancer immune microenvironment.

As one of the most important immune cells in cancer, macrophages have a relatively high expression of immune cells, which can not only regulate the nonspecific immunity, but also influence the specific immunity. In nonspecific immunity, macrophages can kill and clear pathogens through phagocytosis and mediate inflammatory response. In specific immunity, macrophages mainly focus on immune regulation and antigen presentation. Studies have shown that repolarization of macrophages from M2 to M1 could enhance the macrophages’ ability to*kill* cancer cells, including ovarian cancer cells [33–35]. Thus, we investigated the correlation between ALKBH5 and IGF2BP2 with the M2 macrophage markers IL-10 and MRC1, while ALKBH5 showed positively correlation, however, no obvious correlation was observed with IGF2BP2.

Single-cell sequencing has been gaining momentum in recent years. The single-cell sequencing technology involves sequencing and analyzing the genome, transcriptome and epigenome at the single-cell level [36]. Traditional sequencing is carried out on the basis of multicellularity. In fact, what we get is the average value of signals in a cluster of cells, which *eliminates* the information of cell heterogeneity. Single-cell sequencing technology can detect the heterogeneity information that can’t be obtained by hybrid sample sequencing, so it effective in addressing this *issue.* Thus, we analyzed the ALKBH5 and IGF2BP2 expression in immune cells through single-level analysis. The results showed that ALKBH5 is widely expressed in various cells, with the highest up-regulation observed in macrophages not only in ovarian cancer, but also in ovarian tissues. However, the expression of IGF2BP2 was relatively low in macrophages in ovarian cancer microenvironment. Thus, we chose ALKBH5 for further study. Here, we chose THP-1 cell line induced by PMA as the macrophage model, which is widely acknowledged macrophage lineage in many researches. Most of the studies investigating the role of macrophages in human use THP-1 cell line and induced it into macrophage by PMA, which could imitate the status of macrophage in vivo, including in ovarian cancer [34, 37–39]. To validate whether ALKBH5 could promote the M2 polarization of macrophages in vitro, macrophages transfected with ALKBH5 that could overexpress or inhibit its expression were used to evaluate the polarization markers of macrophages through RT-PCR. The results showed that overexpression of ALKBH5 could promote high expression of M2 marker IL-10, ARG-1 and low expression of M1 marker TNF-α, and inhibition of ALKBH5 could promote high expression of M1 marker CD86 with low expression of M2 marker CD163 and CCL22. In fact, many genes and proteins can regulate the polarization of macrophages. However, the function of enzymes that can regulate the m6A methylation in controlling macrophage polarization has not been widely investigated yet. Based on the GO and KEGG analysis, results showed that the biological process of ALKBH5 in ovarian cancer was mostly correlated with immune response, including T cell receptor signaling pathway, inflammatory response, immune response and antimicrobial humoral immune response mediated by antimicrobial peptide. These processes were confirmed as the main processes in immune response in cancer metabolism. What’s more, in KEGG analysis, we *identified* pathways that related to ALKBH5 were Th17 cell differentiation, intestinal immune network for IgA production, NOD-like receptor signaling pathway and NF-κB signaling pathway. Those pathways also showed significantly correlation with immune response. Thus, we speculated that ALKBH5 might participate in regulating the immune response in ovarian cancer microenvironment.

## Conclusion

In conclusion, our study demonstrated that ALKBH5 and IGF2BP2 were significantly up-regulated in M2 macrophages, which not only showed closely correlation with macrophage expression in ovarian cancer, but also correlated with the prognosis of ovarian cancer. In single-cell analysis, we found that ALKBH5 was mainly expressed in macrophages in ovarian cancer. Finally, we verified that ALKBH5 could regulate M2 macrophage polarization in vitro study. Thus, targeting ALKBH5 in macrophages might be a promising target for regulating the immune microenvironment in ovarian cancer, which could further influence the prognosis of ovarian cancer patients.

### Supplementary Information

**Additional file 1: Supplementary Figure 1.** Distinct expression of m6A methylation enzymes in M0 and M2 macrophages. **Supplementary Figure 2.** The expression of ALKBH5 and IGF2BP2 in distinct tissues. **Supplementary Figure 3.** The genes and pathways correlated with ALKBH5 in ovarian cancer. **Supplementary Table 1.** The primers used in the study. **Supplementary Table 2.** The DEGs according to the expression level of ALKBH5 in TCGA datasets

## Data Availability

All the data obtained and/or analyzed during the current study were available from the corresponding authors on reasonable request.
