# Supplementary material for: ALKBH5 modulates macrophages polarization in tumor microenvironment of ovarian cancer
Source: J Ovarian Res. 2024 Apr 18;17:84. doi: 10.1186/s13048-024-01394-4 (PMC11025218; doi:10.1186/s13048-024-01394-4)
Supplement: Supplementary file 1 — Additional file 1: Supplementary Figure 1. Distinct expression of m6A methylation enzymes in M0 and M2 macrophages. Supplementary Figure 2. The expression of ALKBH5 and IGF2BP2 in distinct tissues. Supplementary Figure 3. The genes and pathways correlated with ALKBH5 in ovarian cancer. Supplementary Table 1. The primers used in the study. Supplementary Table 2. The DEGs according to the expression level of ALKBH5 in TCGA datasets [file 13048_2024_1394_MOESM1_ESM.docx]

**Supplementary Figure 1: Distinct expression of m6A methylation enzymes in M0 and M2 macrophages.** (A) Differential expressed genes between M0 and M2 macrophages in GSE35495. (B) Differential expressed genes between M0 and M2 macrophages in GSE36537. (C) Heatmap plots showed differential expression of m6A methylation enzymes between M0 and M2 macrophages in GSE35495 and GSE36537.


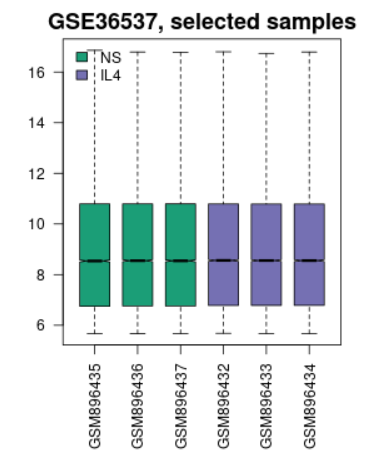

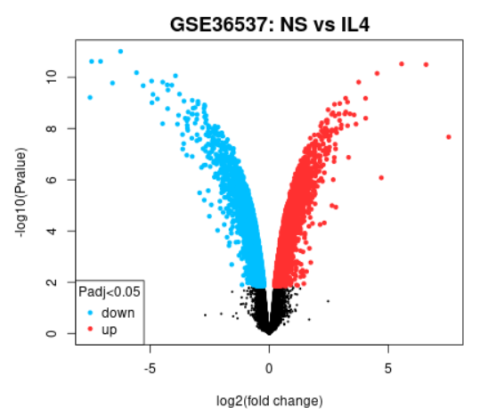

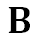

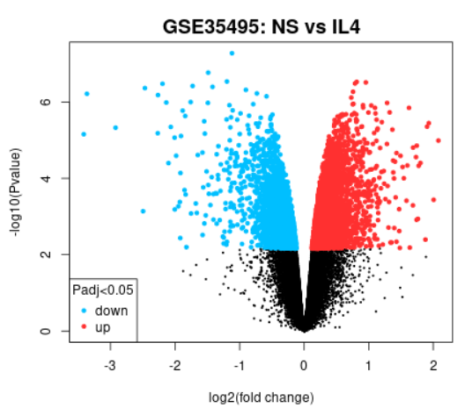

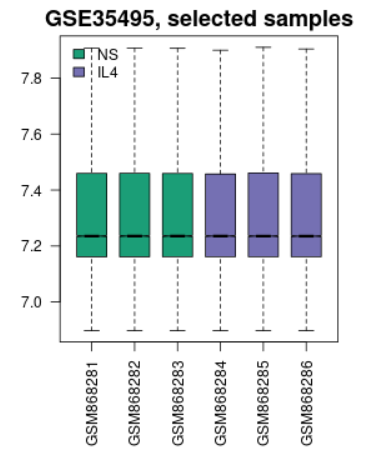

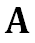

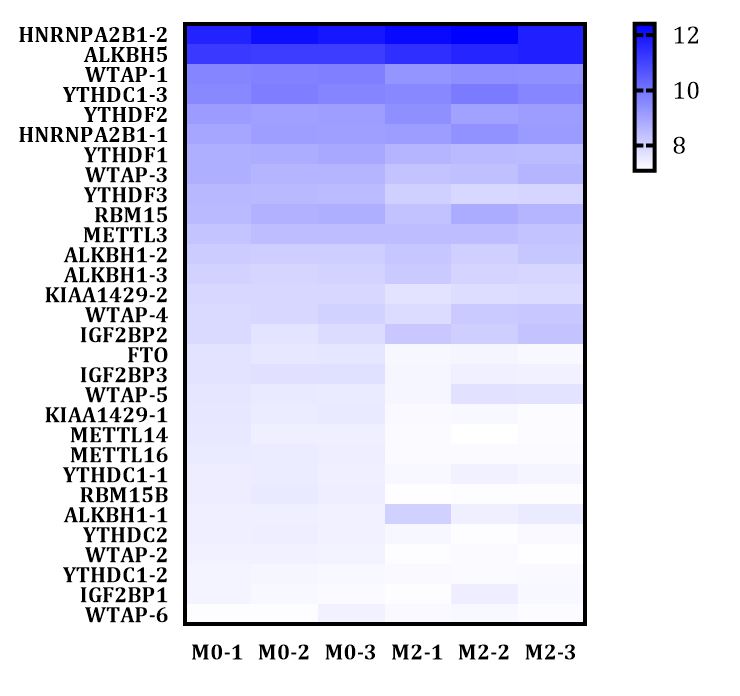

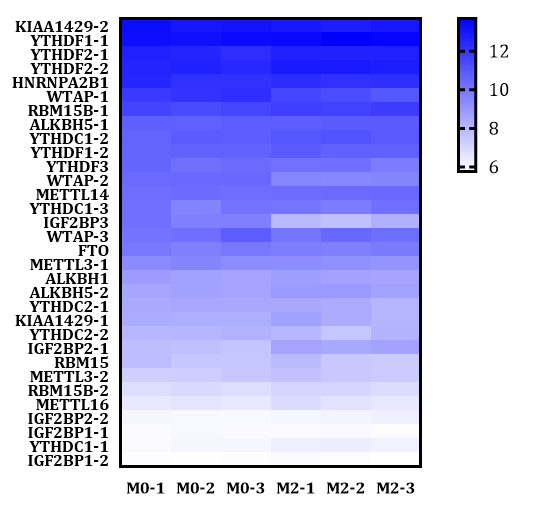

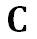


**Supplementary Figure 2: The expression of ALKBH5 and IGF2BP2 in distinct tissues.** (A) The relative expression of ALKBH5 and IGF2BP2 in distinct tissues. The above figure showed the expression of ALKBH5, while the below figure showed the expression of IGF2BP2. (B) The expression of ALKBH5 and IGF2BP2 in distinct immune phenotypes in ovarian cancer.


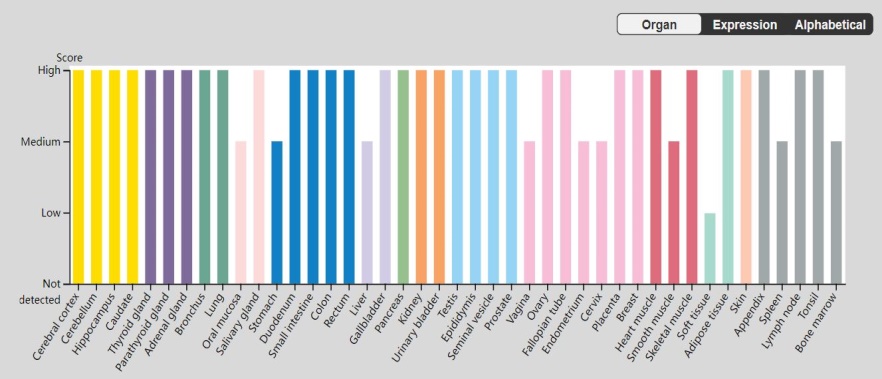

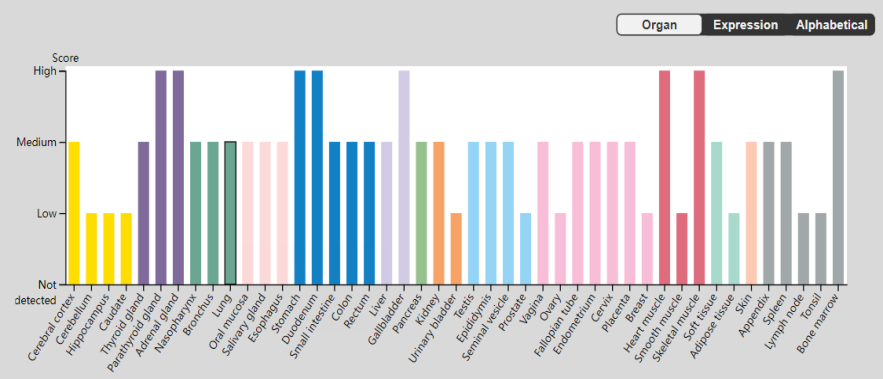

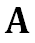

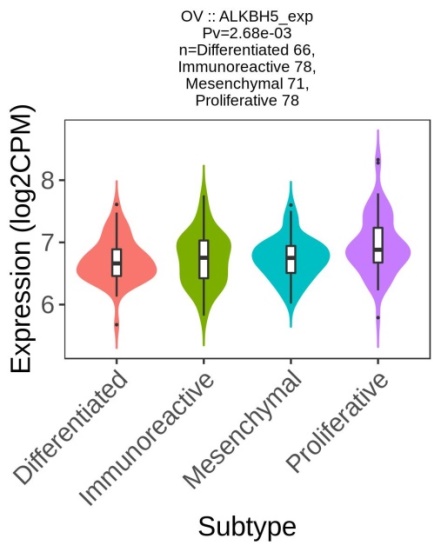

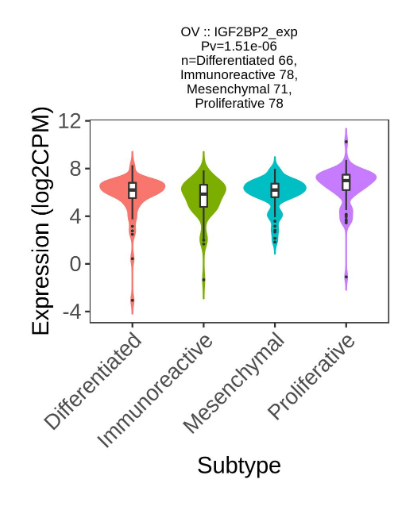

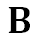


**Supplementary Figure 3:** The genes and pathways correlated with ALKBH5 in ovarian cancer. (A) Top 20 hub genes collected through cytoHubba. (B) The relative pathways of ALKBH5 in ovarian cancer. (C) The relative pathways of IGF2BP2 in ovarian cancer.


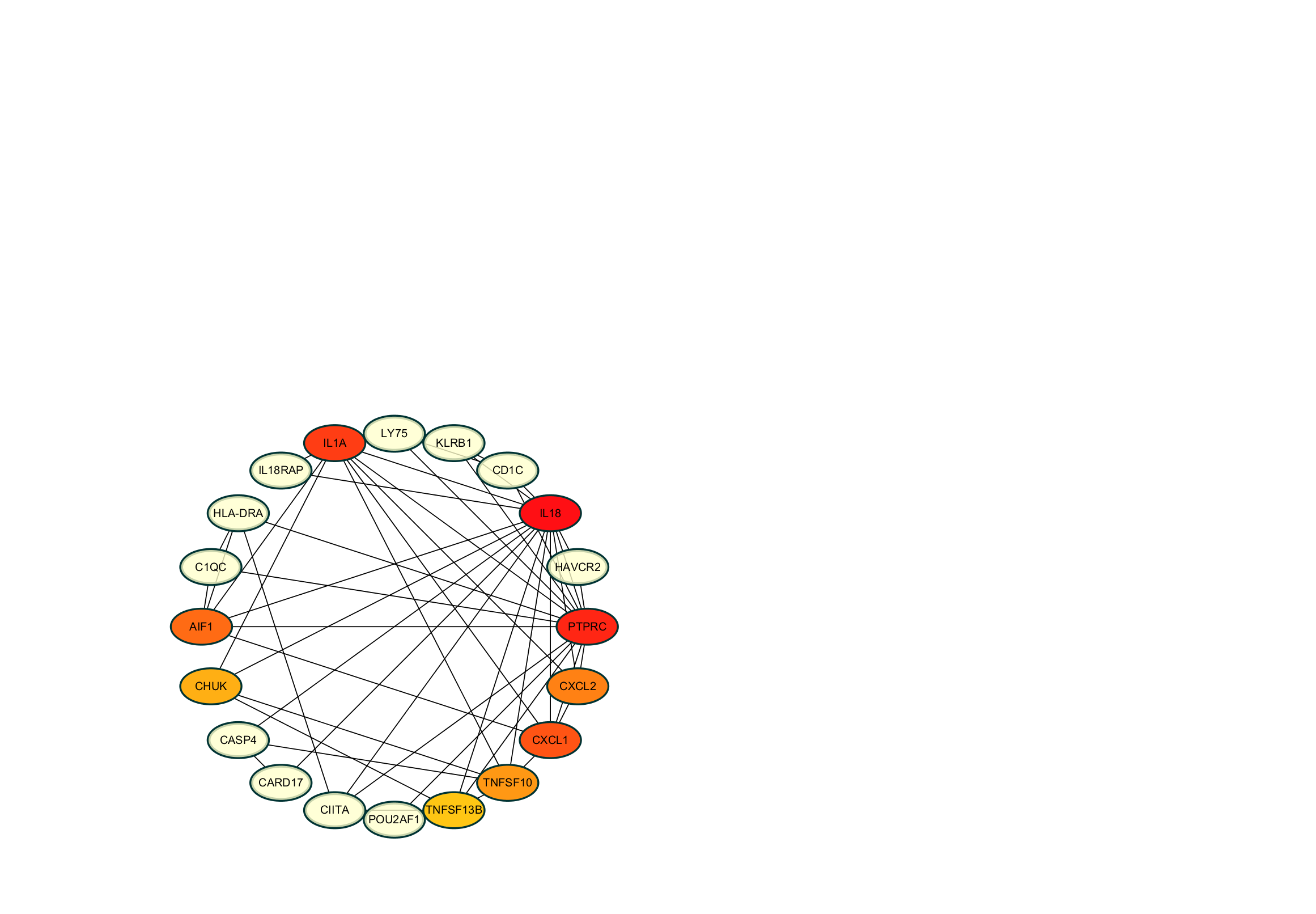

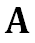

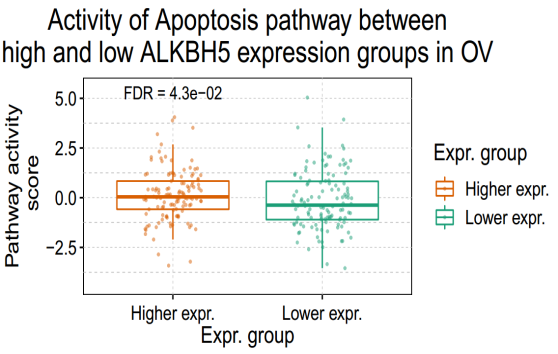

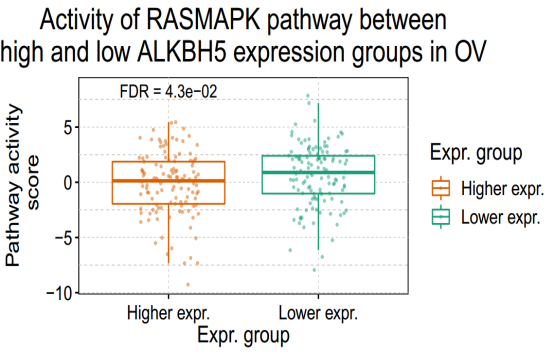

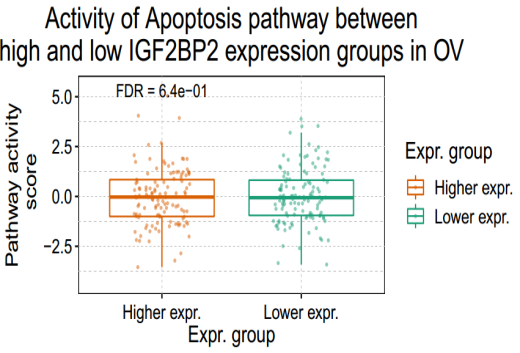

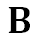

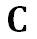

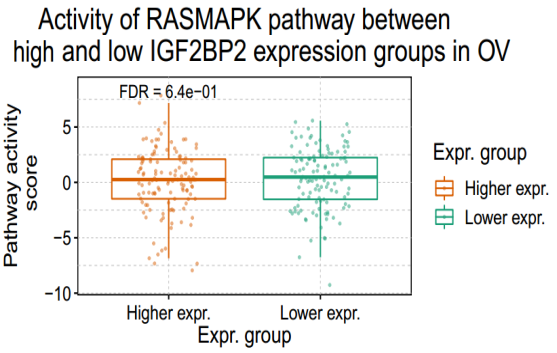


**Supplementary Table 1**  The primers used in the study

| Ontology | ID | Description | Count | P-value | Fold enrichment |
| --- | --- | --- | --- | --- | --- |
| KEGG | Hsa04060 | Cytokine-cytokine receptor interaction | 11 | 0.000662 | 3.688797024 |
| KEGG | Hsa05323 | Rheumatoid arthritis | 6 | 0.002293243 | 6.382376082 |
| KEGG | Hsa05164 | Influenza A | 7 | 0.007076995 | 4.049636286 |
| KEGG | Hsa04061 | Viral protein interaction with cytokine and cytokine receptor | 5 | 0.017545843 | 4.946341463 |
| KEGG | Hsa05321 | Inflammatory bowel disease | 4 | 0.02689861 | 6.087804878 |
| KEGG | Hsa04621 | NOD-like receptor signaling pathway | 6 | 0.036491698 | 3.225874867 |
| KEGG | Hsa04064 | NF-kappa B signaling pathway | 4 | 0.085387502 | 3.804878049 |
| KEGG | Hsa04672 | Intestinal immune network for IgA production | 3 | 0.085719612 | 6.056744649 |
| KEGG | Hsa04659 | Th17 cell differentiation | 4 | 0.09311625 | 3.66395664 |

**Supplementary Table 2**  The DEGs according to the expression level of ALKBH5 in TCGA datasets

| Gene name | logFC | P-Value |
| --- | --- | --- |
| ACSL5 | -0.40668 | 0.001369 |
| ACTN2 | 0.289216 | 0.041228 |
| ADAR | 0.286675 | 7.28E-08 |
| ADHFE1 | -0.28965 | 0.002786 |
| AHR | -0.33362 | 0.000257 |
| AIF1 | -0.32265 | 0.019651 |
| ALS2CR16 | -0.29048 | 0.000281 |
| AOF2 | 0.269509 | 4.35E-05 |
| APOC1 | -0.34553 | 0.008757 |
| ARHGAP26 | -0.40345 | 0.000255 |
| ARMC3 | 0.532874 | 0.020892 |
| ARNT2 | 0.322626 | 0.006304 |
| ATP5B | 0.267363 | 0.000336 |
| ATP7B | 0.327483 | 0.001085 |
| B3GNT3 | -0.39858 | 0.009349 |
| BCAT1 | 0.519459 | 0.017746 |
| BFSP1 | 0.294624 | 0.011539 |
| BMP7 | 0.684428 | 0.00024 |
| C10orf81 | -0.63977 | 0.013976 |
| C17orf60 | -0.44836 | 0.000109 |
| C17orf76 | 0.365164 | 0.00012 |
| C1orf108 | 0.291976 | 0.001503 |
| C1QC | -0.30264 | 0.00246 |
| C20orf11 | 0.265832 | 3.75E-06 |
| C22orf36 | 0.346102 | 0.002133 |
| C3orf21 | 0.296672 | 1.56E-05 |
| C3orf34 | 0.297123 | 0.000865 |
| C6orf154 | 0.450126 | 9.57E-05 |
| C9orf95 | -0.32121 | 0.000152 |
| CA12 | -0.32393 | 0.046204 |
| CASP4 | -0.42924 | 5.9E-08 |
| CBR3 | -0.50407 | 0.000753 |
| CD1C | -0.26625 | 0.022206 |
| CDH18 | 0.774685 | 0.017328 |
| CHRM2 | 0.2759 | 0.026335 |
| CHUK | 0.296827 | 0.000202 |
| CIITA | -0.27823 | 0.008725 |
| CKMT1A | -0.44059 | 0.00129 |
| CLUL1 | 0.293638 | 0.04188 |
| COMTD1 | -0.27161 | 0.008612 |
| CPNE4 | 0.439046 | 0.002658 |
| CPXM1 | 0.483931 | 7.29E-05 |
| CRK | 0.301218 | 3.79E-08 |
| CRYAB | -0.26724 | 0.033209 |
| CSTA | -0.39823 | 0.009579 |
| CTNNA2 | 0.382107 | 0.018123 |
| CTNNBL1 | 0.269062 | 4.85E-07 |
| CTSO | 0.271467 | 0.01868 |
| CXCL1 | -0.59995 | 0.017275 |
| CXCL2 | -0.38466 | 0.029322 |
| CYP39A1 | 0.270055 | 0.028762 |
| DDIT4L | -0.39548 | 0.032243 |
| DEFB1 | -0.35582 | 0.032536 |
| DEPDC6 | -0.38716 | 0.012639 |
| DIRAS3 | 0.283714 | 0.047815 |
| DNAH2 | 0.36632 | 0.000824 |
| DSC3 | 0.320944 | 0.046655 |
| EML1 | 0.290609 | 0.009461 |
| ESM1 | 0.336378 | 0.000202 |
| EYA4 | 0.774783 | 0.000402 |
| FADS1 | 0.290822 | 0.007764 |
| FAM125B | 0.307203 | 9.54E-07 |
| FAM18B | 0.306918 | 4.95E-07 |
| FAM3D | -0.35458 | 0.036119 |
| FBLN2 | 0.321578 | 0.029103 |
| FBXO32 | -0.30073 | 0.033168 |
| FHAD1 | 0.343793 | 0.003862 |
| FOLH1 | 0.39907 | 0.000281 |
| FREM1 | 0.378352 | 0.027242 |
| FSCN1 | 0.329394 | 0.000702 |
| FXYD6 | 0.319256 | 0.008863 |
| GABRE | -0.39174 | 0.011902 |
| GBP4 | -0.36578 | 0.014751 |
| GDF11 | 0.418693 | 0.001092 |
| GDPD2 | 0.331039 | 0.01279 |
| GDPD5 | 0.350121 | 0.000309 |
| GMFG | -0.31209 | 0.001748 |
| GNLY | -0.35451 | 0.047896 |
| GPHB5 | -0.286 | 9.79E-06 |
| GRAMD1C | -0.35559 | 0.001196 |
| GRN | 0.345909 | 5.89E-07 |
| GSPT1 | 0.274977 | 0.005138 |
| HAVCR2 | -0.30519 | 0.008725 |
| HLA-DRA | -0.30259 | 0.009172 |
| HNMT | -0.3621 | 0.000788 |
| HNRPD | 0.26436 | 3.17E-05 |
| HPN | 0.441428 | 0.009312 |
| HRH1 | -0.45243 | 0.000685 |
| IFNE1 | -0.31101 | 0.012784 |
| IGFBP2 | 0.482555 | 0.000354 |
| IGSF9 | 0.267002 | 0.001457 |
| IL15RA | -0.29971 | 0.018542 |
| IL18 | -0.38553 | 0.005153 |
| IL18RAP | -0.30011 | 0.028392 |
| IL1A | -0.37539 | 0.00616 |
| IL27 | -0.26717 | 0.043674 |
| IMAA | -0.3066 | 0.00717 |
| INCA | -0.27471 | 0.005485 |
| KCNH3 | 0.306045 | 0.003288 |
| KCNJ12 | 0.504684 | 7.37E-05 |
| KCNN3 | 0.361839 | 0.000263 |
| KCNS1 | 0.283399 | 0.021148 |
| KIAA0895 | 0.29062 | 0.001007 |
| KIAA1199 | 0.28121 | 0.032889 |
| KIAA1211 | 0.297062 | 0.001627 |
| KIAA1219 | 0.276142 | 3.52E-07 |
| KLRB1 | -0.38407 | 0.032536 |
| KLRF1 | 0.304275 | 0.046204 |
| KRT4 | -0.38302 | 0.02503 |
| KRTCAP3 | -0.30178 | 0.001762 |
| LEAP2 | -0.27072 | 0.004297 |
| LGR5 | 0.447667 | 0.019445 |
| LOC129881 | 0.36852 | 0.027634 |
| LOC130951 | 0.270647 | 0.01298 |
| LOC400566 | 0.52873 | 2.98E-08 |
| LOC440356 | 0.421382 | 0.005153 |
| LOH11CR2A | -0.3433 | 0.00068 |
| LPHN1 | 0.341931 | 0.000165 |
| LRRC43 | 0.348801 | 0.007035 |
| LRRIQ1 | 0.368415 | 0.004915 |
| LSM14B | 0.331476 | 2.47E-07 |
| LY75 | -0.31177 | 0.013177 |
| LYPD2 | -0.3568 | 0.040096 |
| LYPLA2 | 0.268675 | 9.24E-05 |
| LYSMD2 | -0.27227 | 0.005707 |
| MINPP1 | 0.291595 | 0.004808 |
| MRFAP1 | 0.346139 | 2.98E-08 |
| MS4A6A | -0.27216 | 0.0385 |
| MST1R | -0.30391 | 0.009176 |
| MYBL2 | 0.352259 | 8.2E-05 |
| MYCL1 | 0.3277 | 0.01015 |
| NACAD | 0.280297 | 0.006239 |
| NAP1L2 | 0.352242 | 0.020781 |
| NAP1L3 | 0.286093 | 0.032129 |
| NCOA6 | 0.264138 | 1.21E-05 |
| NPAS3 | 0.347598 | 0.02807 |
| NXF5 | 0.35176 | 0.015314 |
| NXNL2 | -0.37195 | 0.013637 |
| OSR2 | 0.308861 | 0.0426 |
| PAM | 0.292 | 0.008181 |
| PANK1 | 0.420709 | 5.8E-05 |
| PCOLCE2 | 0.31296 | 0.020028 |
| PDE1A | -0.43874 | 0.012089 |
| PEMT | 0.319271 | 2.83E-09 |
| PIGT | 0.408199 | 1.4E-09 |
| PIGU | 0.309491 | 1.09E-07 |
| PIP4K2C | 0.371446 | 1.78E-09 |
| PKIB | -0.37287 | 0.012226 |
| PLEKHB1 | 0.346522 | 6.32E-05 |
| PNMA1 | 0.33768 | 8.6E-06 |
| POU2AF1 | 0.446066 | 0.019228 |
| PPP4R1 | 0.277501 | 3.61E-05 |
| PRRG4 | -0.3667 | 0.000223 |
| PSMB10 | -0.27876 | 0.002222 |
| PTPRC | -0.28551 | 0.041228 |
| PVRL3 | 0.575107 | 4.37E-05 |
| RAB17 | -0.32529 | 0.000399 |
| RAMP1 | -0.32248 | 0.046204 |
| RARRES3 | -0.40682 | 0.000399 |
| RBM38 | 0.355706 | 2.92E-07 |
| RP4-747L4.3 | 0.289422 | 0.002089 |
| RPN2 | 0.297167 | 2.83E-09 |
| RPS18 | -0.26535 | 0.001 |
| RPSA | -0.30401 | 1.36E-05 |
| SAA4 | -0.34641 | 0.021496 |
| SALL2 | 0.381289 | 0.000696 |
| SCAMP3 | 0.276701 | 1.07E-08 |
| SCML1 | -0.30841 | 0.001284 |
| SEMA3E | 0.46023 | 0.047 |
| SERPINB1 | -0.27649 | 0.00755 |
| SGMS1 | -0.29199 | 0.001114 |
| SKAP1 | -0.29558 | 0.016726 |
| SLC10A4 | 0.621442 | 1.74E-05 |
| SLC35C2 | 0.273945 | 2.92E-07 |
| SLC39A7 | 0.301934 | 0.000365 |
| SMARCB1 | 0.270737 | 5.64E-05 |
| SNCG | -0.38782 | 0.026247 |
| SNRPN | -0.3279 | 0.005237 |
| SNX17 | 0.273054 | 0.000903 |
| SOX4 | 0.281206 | 0.004222 |
| SPAG6 | 0.423144 | 0.006239 |
| SPATS2 | 0.282051 | 3.74E-05 |
| ST6GALNAC3 | 0.265786 | 0.015168 |
| STK33 | 0.26442 | 0.039843 |
| STRC | -0.27419 | 0.00724 |
| TACSTD2 | -0.26644 | 0.03009 |
| TEPP | -0.27527 | 0.000399 |
| TGIF2 | 0.394196 | 9.54E-07 |
| TM4SF4 | 0.336959 | 0.045881 |
| TMC5 | -0.33763 | 0.003724 |
| TMEM40 | -0.3707 | 0.001554 |
| TMEM45A | 0.428279 | 0.001027 |
| TNFAIP8 | -0.28372 | 0.009433 |
| TNFAIP8L2 | -0.31759 | 0.005339 |
| TNFSF10 | -0.41398 | 0.003021 |
| TNFSF13B | -0.36868 | 0.00939 |
| TOMM34 | 0.299213 | 1.38E-06 |
| TPBG | -0.30645 | 0.039829 |
| TRAT1 | -0.26889 | 0.023482 |
| TRPM8 | -0.43422 | 0.003086 |
| TSR1 | 0.275186 | 2.25E-06 |
| TTYH1 | 0.536318 | 0.002576 |
| TXNDC3 | -0.42797 | 0.00671 |
| TXNRD1 | 0.343503 | 3.52E-07 |
| UCP1 | 0.297976 | 0.00035 |
| WBSCR17 | 0.481075 | 0.000395 |
| ZCCHC12 | 0.306153 | 0.019228 |
| ZNF286A | 0.279114 | 0.000153 |
| ZNF423 | 0.315898 | 0.023913 |
| ZNF502 | -0.30565 | 0.00918 |
| ZNF594 | 0.265884 | 0.000175 |
